# A Cross-Sectional Study: Nutritional Polyamines in Frequently Consumed Foods of the Turkish Population

**DOI:** 10.3390/foods3040541

**Published:** 2014-10-09

**Authors:** Nihal Buyukuslu, Hilal Hizli, Kubra Esin, Muazzez Garipagaoglu

**Affiliations:** Department of Nutrition and Dietetics, School of Health Sciences, Istanbul Medipol University, Beykoz/Istanbul, 34810, Turkey; E-Mails: hhizli@medipol.edu.tr (H.H.); kesin@medipol.edu.tr (K.E.); mgaripagaoglu@medipol.edu.tr (M.G.)

**Keywords:** polyamine, putrescine, spermidine, spermine, daily intake, diet, health

## Abstract

Putrescine, spermidine and spermine are the most abundant polycationic natural amines found in nearly all organisms. They are involved in regulation of gene expression, translation, cell proliferation and differentiation. They can be supplied by the endogenous synthesis inside the cell or by the intake from exogenous sources. There is a growing body of literature associated with the effects of bioactive amines on health and diseases, but limited information about polyamine content in foods is available. In the present study, the polyamine content of frequently consumed foods in a typical Turkish diet was estimated for adults, including tea, bread and yoghurt. The estimation of daily intake was defined as 93,057 nmol/day putrescine, 33,122 nmol/day spermidine, 13,685 nmol/day spermine. The contribution of foods to daily intake was: dairy products (47.32%), vegetables and grains (21.09%) and wheat products (12.75%).

## 1. Introduction

Putrescine, spermidine, and spermine are the polyamines that have been of main interest among foods due to their biological activities that have beneficial health effects. They are synthesized in all prokaryotic and eukaryotic cells and are known to play some essential roles in cell proliferation, regeneration and differentiation [1]. The metabolic requirement for polyamines is high in rapidly growing tissues, such as normal growth and development, and in tumors [2,3]. Besides many beneficial effects, it was shown that the level of polyamines in cells is also associated with diseases such as cancers and chronic diseases. Since polyamine is involved in the progression of cancer [4], inhibiting polyamine synthesis and reducing its intake through food may have beneficial effects [5].

The body pool of polyamines is supplied by endogenous or *de novo* biosynthesis, intestinal microorganisms, and exogenous sources through the diet [6]. The amount of external dietary polyamines is higher than the endogenous biosynthesis [1]. Nonetheless, dietary polyamines have been thought to support metabolism maintaining optimal health. For instance, they have a potential role in growth and development of digestive system [7]. Intracellular production of polyamines and their concentration in tissues and organs decreases with aging. Therefore, intake of exogenous polyamines was shown to be beneficial in the treatment of some geriatric diseases [8,9]. In the meantime, Binh reported a possible role for the food polyamines that are abundant in the Mediterranean diet in prolonging human life [10]. However, limited information about the polyamine content of food is available to assess dietary polyamine intake and subsequently have dieticians design proper menus. In addition, dietary amines are classified in two categories according to their synthesis; natural polyamines which are formed during *de novo* polyamine biosynthesis, and biogenic amines that are produced by decarboxylation of amino acids. Among natural polyamines, putrescine belongs to both categories. The daily putrescine requirement can be met from either natural or biogenic sources. Inappropriate storage and processing conditions result in biogenic amin formation in foods. Therefore, especially in the case of cancer patients, the daily polyamine intake should be carefully evaluated.

Polyamine contents in foods vary widely between and even within food types [1,11]. This might be due to the origin, processing, storage conditions, seasonal variation and different methodological applications of foods. Thus far, studies on polyamine concentration in food and on the estimation of dietary polyamine were based on the analyses of polyamines in each food by defining the daily intake of foods from a nutrient database and calculating daily intake of polyamines. An inadequate polyamine database seems to be the main limitation for daily estimation of polyamine intake.

Dietary habits in Turkey vary according to region, culture, socioeconomic status, gender and age of the individual. A recent cohort study in Turkey [12] indicated that the most frequently consumed foods were black tea, white bread types, cheese, sunflower oil, sugar, honey, jam and pekmez. The major percentage of energy came from bread. Cheese and yogurt were the most frequently used milk products. Fresh fruits and vegetables were widely consumed throughout the year. Oil and fat consumption showed regional variations. Most frequently used oils were sunflower and olive.

Although there are few studies defining polyamine content in fermented foods such as cheese and meat products, the polyamine content in many widespread foods in Turkey do not exist in a nutrient database. This study aims to identify the main sources of polyamines in frequently consumed foods and to estimate daily putrescine, spermine, and spermidine intake.

## 2. Experimental Section

### 2.1. Study Population

In this cross-sectional study, the daily intake of foods of 1218 subjects (45.1% of male; 54.9% of female) was examined by trained interviewers. The subjects were randomly selected from different regions in Turkey. The highest prevalence of distribution among regions was observed in the Mediterranean (42.0%), followed by Central Anatolia (17.5%), Southeastern Anatolia (15.8%), Eastern Anatolia (9.7%), and Marmara (8.7%); it was lowest in the Black Sea region (6.3%).

The mean age and the mean body mass index (BMI) were 40.02 ± 19.15 and 26.08 ± 4.84, respectively. The study took place between July and October 2011.

### 2.2. Daily Intake of Food

Nutritional information of subjects was collected via a 24 h dietary recall. The quantification of the food and drink was estimated rather than weighed. The interviewers converted these estimates into weights that could be used to calculate food and nutrient intake.

The amounts of daily consumption were registered in grams or milliliters. The dietary records were entered into a computerized nutrient analysis programme BeBis (version 7.2, Pasific Compony, Istanbul, Turkey). Daily intake of foods was calculated as g/person/day. Only in the 81 foods for which the polyamine values were determined have been included in the present study in order to focus on the polyamine content.

Foods were classified as vegetables and grains, fruits, meat products, dairy products, eggs, tea, wheat products, nuts and dried fruits and sweets to define the distribution of polyamines. Foods were ranked from highest to lowest daily consumption of each food item for each group in Table 1.

### 2.3. Database Development for Polyamine in Foods

Data on content of polyamines in different foods was collected through an extensive literature search using PubMed, Web of Science and the Turkish Academic Network and Information Center (ULAKBIM). “Polyamine”, “putrescine”, “spermidine”, “spermine”, “daily intake” and “nutrition” were the key words for the search.

The polyamine contents in foods vary widely between and even within food types. In our research, when an individual food item was obtained from several references or a single reference reported multiple analyses for the same food group, the mean value was calculated. Since the values for polyamine content in some of the studies were reported in nmol/g of food, they were converted into mg/kg, using the appropriate equation (mol = mass/molecular weight). The molecular weights for polyamines were 88.15 g/mol putrescine, 145.25 g/mol spermidine and 202.34 g/mol spermine. The values of polyamines (mg/kg) in food from other publications were placed in Table 1 with references.

**Table 1:** Daily polyamine intake based on the reference values in the most frequently consumed foods in the Turkish population.

Foods	A—Daily Polyamine Intake in Turkish Population					B—Reference Values			
	Daily Intake of Food (g-mL/Person/Day) ^a^	Daily Intake of Each Polyamine (mg-mL/Person/Day) ^a^			Daily Intake of Total Polyamine (mg-mL/Person/Day) ^a^	Polyamines in Foods(mg-mL/kg) ^a^			References
		Putrescine ^b^	Spermidine ^c^	Spermine ^d^		Putrescine ^b^	Spermidine ^c^	Spermine ^d^	
**Vegetables and Grains**									
Rice ^e^	62.30	0.047	0.121	0.287	0.455	0.76	1.95	4.6	[1,13,14]
Tomato ^e^	61.94	0.365	0.164	0.000	0.529	5.9	2.65	nd ^f^	[1,13,14,15]
Lentil soup	44.31	0.146	0.000	0.328	0.474	3.3	-	7.4	[16]
Cucumber ^e^	38.50	0.307	0.326	0.020	0.653	7.98	8.46	0.53	[1,14,15,17]
Green pepper ^e^	9.18	0.572	0.099	0.041	0.712	62.35	10.75	4.5	[14,18]
Eggplant ^e^	4.46	0.110	0.020	0.002	0.132	24.57	4.4	0.5	[14,15]
Onion ^e^	4.39	0.010	0.036	0.007	0.053	2.34	8.3	1.57	[1,14,15,19]
Chickpea	1.68	0.004	0.000	0.002	0.006	2.6	0.1	1.2	[15]
Potato chips ^e^	1.57	0.022	0.027	0.004	0.053	13.84	17.12	2.52	[14,16]
Okra	1.39	0.030	0.026	0.000	0.056	21.7	18.6	Nd ^g^	[9]
Tomato puree	1.18	0.031	0.010	0.002	0.043	25.9	8.4	2.1	[20]
Carrot ^e^	0.72	0.004	0.005	0.001	0.010	6.03	6.42	2,54	[1,13,14,17,18,21]
Potato ^e^	0.58	0.006	0.008	0.001	0.015	10.4	14.02	1.85	[1,13,14,17,18,19,21]
Cabbage ^e^	0.57	0.010	0.006	0.002	0.018	17.58	10.39	2.91	[1,14,19,21]
Lettuce ^e^	0.45	0.005	0.008	0.000	0.013	10.08	18.83	0.4	[1,14,17,19]
Green peas ^e^	0.34	0.002	0.022	0.018	0.042	5.7	65.2	52.5	[14,22]
Cauliflower ^e^	0.23	0.001	0.006	0.001	0.008	4.67	28.1	4.25	[1,17,21]
Ketchup ^e^	0.20	0.006	0.001	0.001	0.008	32.42	5.15	2.7	[14,20]
Spring onion	0.19	0.005	0.003	0.000	0.008	24.5	17	0.2	[9]
Broccoli ^e^	0.14	0.001	0.005	0.001	0.007	5.75	32.39	9.3	[14,17,21]
Mushroom	0.14	0.001	0.012	0.000	0.013	4	88.6	3.4	[14]
Celeriac	0.10	0.001	0.003	0.000	0.004	6.1	26.7	0	[21]
Celery	0.10	0.002	0.001	0.000	0.003	17.1	14.2	3.8	[14]
Maize ^e^	0.06	0.003	0.002	0.000	0.005	50.7	32.1	1.4	[9,14]
Garlic	0.05	0.000	0.001	0.000	0.001	2.3	11.1	5.8	[14]
Dill	0.03	0.000	0.001	0.000	0.001	12.7	29.2	8.7	[9]
Spinach ^e^	0.02	0.000	0.000	0.000	0.000	8.43	16.03	2.1	[14,19,20]
Mayonnaise ^e^	0.18	0.000	0.000	0.000	0.000	0.53	1.6	0.3	[14,15]
**Fruits**									
Watermelon	69.70	0.000	0.084	0.000	0.084	nd ^f^	1.2	nd ^f^	[9]
Peach ^e^	44.32	0.023	0.194	0.114	0.331	0.52	4.38	2.57	[9,15]
Melon	31.70	0.013	0.371	0.000	0.384	0.4	11.7	nd ^f^	[9]
Apple ^e^	28.40	0.153	0.045	0.001	0.199	5.39	1.6	0.05	[1,9,14,20]
Grapes	27.10	0.003	0.002	0.000	0.005	0.1	0.06	0.01	[23]
Cherry	5.49	0.009	0.009	0.004	0.022	1.6	1.6	0.8	[14]
Fig	5.69	0.013	0.030	0.000	0.043	2.2	5.2	nd ^f^	[9]
Pear ^e^	4.98	0.061	0.007	0.000	0.068	12.2	1.5	nd ^f^	[1,15]
Banana ^e^	3.04	0.042	0.026	0.003	0.071	13.8	8.55	1.05	[14,15]
Tangerine ^e^	1.39	0.100	0.003	0.000	0.103	72.28	1.87	0.2	[14,17]
Orange ^e^	0.67	0.066	0.003	0.000	0.069	98.28	3.75	0.45	[1,13,14,17]
Strawberry	0.67	0.001	0.001	0.000	0.002	1	2	0.4	[14]
Lemon (lime)	0.58	0.024	0.003	0.001	0.028	41	5	1.8	[9]
Kiwi	0.22	0.000	0.001	0.000	0.001	1.2	5.4	1.5	[15]
Pineapple	0.15	0.000	0.001	0.000	0.001	0.7	4	2.2	[15]
Grapefruit	0.08	0.005	0.001	0.000	0.006	62.1	7.3	nd ^f^	[20]
**Tea**									
Black tea ^e^	322.2	0.750	2.430	0.050	3.230	0.750	2.430	0.050	[13,24]
**Wheat products**									
Bread, white ^e^	201.4	0.282	1.299	0.624	2.205	1.4	6.45	3.1	[1,15]
Pasta	21.64	0.010	0.072	0.039	0.121	0.44	3.34	1.82	[14]
Pasta, cooked	14.81	0.015	0.107	0.160	0.282	1	7.2	10.8	[1]
Bread, whole grain ^e^	5.04	0.013	0.090	0.032	0.135	2.53	17.77	6.3	[1,15,17]
Flour ^e^	0.91	0.002	0.007	0.003	0.012	2.16	7.27	3.36	[13,14]
**Meat and fish products**									
Chicken breast, cooked ^e^	4.14	0.000	0.106	0.224	0.330	0	25.5	54.1	[20,25]
Chicken thigh ^e^	4.31	0.002	0.037	0.117	0.156	0.4	8.7	27.1	[20,25]
Chicken ^e^	1.94	0.007	0.012	0.108	0.127	3.42	6.25	55.74	[1,13,14]
Minced meat, beef ^e^	1.59	0.010	0.059	0.054	0.123	6.45	37.05	33.85	[1,17]
Lamb meat	1.46	0.001	0.007	0.069	0.077	1	5	47.1	[13]
Salami	1.44	0.001	0.004	0.013	0.018	0.5	3	9	[15]
Beef meat ^e^	1.22	0.005	0.009	0.040	0.054	4.07	7.55	32.95	[1,13,17,26]
Chicken grilled ^e^	1.14	0.002	0.020	0.051	0.073	2	17.3	44.4	[20,25]
Chicken breast ^e^	1.12	0.000	0.007	0.030	0.037	0.4	6.25	27	[20,25]
Sausages	1.09	0.015	0.007	0.027	0.049	14.2	6.1	25	[1]
Beef liver	0.58	0.001	0.004	0.114	0.119	1	6.8	197	[9]
Tuna, canned	0.17	0.000	0.001	0.003	0.004	1.22	5.75	18.7	[27]
Salmon ^e^	0.16	0.000	0.001	0.001	0.002	2.59	4.07	5.72	[9,14,17]
Tuna (in oil)	0.11	0.000	0.000	0.000	0.000	0.01	1.4	0	[27]
**Nuts and dry fruits**									
Hazelnut	1.39	0.006	0.029	0.009	0.044	4.2	21	6.5	[15]
Almond	0.24	0.000	0.001	0.003	0.004	1.6	6	13.5	[14]
Raisin	0.52	0.000	0.000	0.000	0.000	0.1	0.4	0.2	[15]
**Sweets**									
Honey	2.36	0.002	0.000	0.000	0.002	0.7	0.1	nd ^f^	[15]
Strawberry marmalade	0.97	0.001	0.002	0.000	0.003	1.4	2	0.4	[15]
Jam	0.48	0.001	0.001	0.000	0.002	1.2	2.2	–	[1]
Apricot marmalade	0.45	0.000	0.001	0.000	0.001	1	1.6	nd ^f^	[15]
Prune marmalade	0.44	0.002	0.001	0.000	0.003	4.6	2	0.4	[15]
Chocolate ^e^	0.23	0.000	0.001	0.000	0.001	0.38	2.19	1.34	[14,15]
Cacao	0.01	0.000	0.000	0.000	0.000	0.26	0.44	0.2	[14]
**Egg**									
Egg ^e^	20.70	0.007	0.012	0.011	0.030	0.32	0.58	0.53	[13,14,16]
**Dairy products**									
Yoghurt ^e^	92.9	0.019	0.035	0.040	0.094	0.2	0.38	0.43	[17,23]
Cheese, white ^g^	37.05	5.039	0.000	0.000	5.039	136	–	–	[28]
Cheddar, fresh ^e^	4.22	0.035	0.006	0.003	0.044	8.31	1.39	0.61	[14,15,29,30,31]
Milk	3.49	0.000	0.000	0.000	0.000	0.013	0.086	–	[32]
Milk, semi-skimmed	3.49	0.001	0.002	0.001	0.004	0.2	0.5	0.3	[1]
Milk, goat ^e^	2.65	0.090	0.002	0.001	0.093	33.87	0.6	0.3	[15,32]

^a^ Solid food items in mg, liquid food items in mL; ^b^ molecular weight of putrescine is 88.15 g/mol; ^c^ molecular weight of spermidine is 145.25 g/mol; ^d^ molecular weight of spermine is 202.34 g/mol; ^e^ the mean value of food item (section B) was calculated based on the references given in section A; ^f^ nd: not determined; ^g^ there were several polyamine concentrations for various cheeses. We included the result from the most consumed “white cheese” in Turkey.

### 2.4. Daily Polyamine Intake in Foods

The concentrations of putrescine, spermidine and spermine in foods were reported in [1,9,10,13,14,15,17,18,19,20,21,22,23,25,26,27,28,29,30,31,33,34]. Average daily intakes of putrescine, spermidine and spermine in mg per person per day were calculated; the sum of these three measures yielded the total polyamine intake (Table 1). Foods were categorized as mentioned in Section 2.2. The contribution of foods to the total daily polyamine intake was identified for each group. Based on average values of polyamines that were given for each 100 g of the food item, the daily putrescine, spermidine and spermine intake from the foods were calculated in nmol per person.

### 2.5. Statistical Analyses

All statistical analyses were performed by using the Statistical Package for Social Sciences (SPSS) version 18.0 (SPSS Inc., Chicago, IL, USA). The values for age and BMI were expressed as means ± standart deviation (SD). Polyamine contents in the individual food items were characterized by arithmetic mean value.

## 3. Results

### 3.1. Polyamine Database

Polyamine concentration of foods was taken from the literature. For each food, the reference where the polyamine values were taken from was denoted on the references column in Table 1. The mean value was calculated if there was more than one value for any food item. The concentrations of polyamines were shown in mg per kg food in Table 1. Rice, tomato, tomato puree, ketchup, lentil soup, cucumber, green pepper, eggplant, onion, spring onion, chickpea, potato, okra, carrot, cabbage, lettuce, green peas, cauliflower, broccoli, mushroom, celery, maize, garlic, dill, spinach were placed in the vegetables and grains group. The highest putrescine per kg food was green pepper (62.35 mg), followed by maize (50.7 mg) and ketchup (32.42 mg); the lowest was rice (0.76). Spermidine concentration in foods was as follows: mushroom (88.6 mg/kg), green peas (65.2 mg/kg), and broccoli (32.39 mg/kg). High spermine-containing foods were: green peas (52.5 mg/kg), broccoli (9.3 mg/kg), and dill (8.7 mg/kg), respectively. Among fruits: watermelon, peach, melon, apple, grapes, cherry, fig, pear, banana, tangerine, orange, strawberry, lemon, kiwi, pineapple and grapefruit were placed in the list. The foods including three high-valued polyamines were: orange (98.28 mg), tangerine (72.28 mg), and lemon (41.00 mg) for putrescine; melon (11.7 mg), banana (8.55 mg), and grapefruit (7.3 mg) for spermidine; peach (2.57 mg), pineapple (2.2 mg), and lemon (1.8 mg) for spermine. Tea is the most frequently consumed drink (322.2 mL/person/day) and white bread is a commonly consumed food (201.4 g/person/day) in Turkey. In general, spermidine concentrations of wheat products were higher than putrescine and spermine levels. Poultry, red meat and meat products such as sausages and salami were grouped as meat products. The highest concentration was spermine (4206 nmol/day), the second was spermidine (1886 nmol/day) and the lowest was putrescine (522 nmol/day) in meat products. Tuna (canned and in oil) and salmon were included in the meat products category since the daily frequency intake was higher than other types of fish. Hazelnut, almond and raisin were in the group of nuts and dry fruits. Honey, marmalade, jam, chocolate and cacao were placed in the sweets group. Yoghurt, cheese and milk were grouped as dairy products. Among dairy products, milk and yoghurt were not rich in polyamines, but several types of cheeses had high levels of polyamines due to the fermentation. The polyamine levels in fresh cheeses were lower than ripened cheeses. Our questionnaire concluded that the most frequently consumed cheese among the Turkish population is white cheese; it was therefore the only type of cheese included to the study. The levels of putrescine, spermidine, and spermine in eggs were 0.32 mg/kg, 0.58 mg/kg and 0.53mg/kg, respectively. All the data obtained from published studies were shown in Table 1, section B.

### 3.2. Frequently Consumed Foods in Turkey

Data from our survey exposed the most frequently consumed foods in Turkey. We picked the ones which have published polyamine values. Foods were listed according to the daily intake of gram food per person per day in Table 1. In g/person/day, rice (62.3), tomato (61.94), and lentil soup (44.31) were the most highly consumed from the vegetables and grains group, whereas watermelon (69.70), peach (44.32) and melon (31.70) were the most consumed fruits. Chicken was consumed more than red meat and fish. Due to lack of polyamine ingredients, most common sweets were not included in the list. Among the sweets, honey intake (2.36 g/person/day) was more than marmalade and jam. Yoghurt (92.9 g/person/day) and cheese consumptions (37.05 g/person/day for ripened; 4.22 g/person/day for fresh) were found higher than daily milk intake. The 15 most frequently consumed foods, from the highest to lowest in g/person/day, were: black tea (322.20), white bread (201.40), yoghurt (92.90), watermelon (69.70), rice (62.30), tomato (61.94), peach (44.32), lentil soup (44.31), cucumber (38.50), ripened cheese (37.05), melon (31.70), apple (28.40), grapes (27.10), pasta (21.64) and egg (20.70).

### 3.3. Daily Polyamine Intake of Frequently Consumed Foods

The calculated values of polyamine in foods are listed in Table 1. The results are given as mg/person/day. The contribution of polyamines in the top five foods of daily intake (mg/person/day) was as follows: ripened cheese (5.039), black tea (0.750), green pepper (0.572), tomato (0.365) and cucumber (0.282) for putrescine; black tea (2.430), white bread (1.299), melon (0.371), peach (0.194) and tomato (0.164) for spermidine; white bread (0.624), lentil soup (0.328), rice (0.287), chicken breast (0.224) and pasta (0.160) for spermine.

Daily intake of polyamines in food groups among the Turkish population was shown in Table 2. The daily putrescine intake came from dairy products (65.48%), vegetables and grains (20.63%), fruits (6.24%), wheat products (3.91%), black tea (2.95%), meat products (0.56%) and eggs (0.09%). The main spermidine sources were wheat products (32.72), vegetables and grains (19.00%), black tea (16.28%), fruits (16.19%), dairy products (9.12%), meat products (5.70%) and eggs (0.25%). The main spermine sources were wheat products (30.99%), vegetables and grains (25.97%), dairy products (6.32%), and fruits (4.55%). Total daily intake of polyamines per person were 93,057 nmol/day putrescine, 33,122 nmol/day spermidine, and 13,685 nmol/day spermine among the most frequently consumed foods in the Turkish population. The main dietary sources for the total polyamines were dairy products (47.32%), vegetables and grains (21.09%) and wheat products (12.75%). Cheese was the primary food item among dairy products contributing to putrescine intake (5.371 mg/person/day) and also to total intake of polyamines.

**Table 2:** Daily intake of polyamines in food groups among the Turkish population.

Foods	Daily Intake of Food Group		Daily Intake of Polyamines										
			Putrescine ^a^			Spermidine ^b^			Spermine ^c^			Total ^d^	
	%	g/person/day	%	mg/person/day	nmol/person/day	%	mg/person/day	nmol/person/day	%	mg/person/day	nmol/person/day	%	nmol/person/day
Tea	26.55	322.20	2.95	0.242	2745	16.28	0.783	5391	0.58	0.016	79	4.95	6725
Wheat products	20.09	243.80	3.91	0.321	3642	32.72	1.574	10,839	30.99	0.858	4240	12.75	17,328
Vegetables and grains	19.35	234.82	20.63	1.692	19,195	19.00	0.914	6293	25.97	0.719	3553	21.09	28,662
Fruits	18.47	224.18	6.24	0.512	5808	16.19	0.779	5363	4.55	0.126	623	7.74	10,525
Dairy products	11.56	140.31	65.48	5.371	60,931	9.12	0.439	3022	6.32	0.175	865	47.32	64,306
Eggs	1.71	20.70	0.09	0.007	79	0.25	0.012	83	0.40	0.011	54	0.16	214
Meat products	1.69	20.47	0.56	0.046	522	5.70	0.274	1886	30.73	0.851	4206	5.69	7735
Sweets	0.41	4.94	0.07	0.006	68	0.10	0.005	34	0.04	0.001	5	0.07	100
Nuts and dried fruits	0.18	2.15	0.07	0.006	68	0.64	0.031	213	0.43	0.012	59	0.22	304
Total ^e^	–	1213.57	–	8.203	93,057	–	4.811	33,122	–	2.769	13,685	–	135,899

^a^ Molecular weight of putrescine is 88.15 g/mol; ^b^ molecular weight of spermidine is 145.25 g/mol; ^c^ molecular weight of spermine is 202.34 g/mol; ^d^ total daily intake of putrescine, spermidine and spermine for each food group; ^e^ daily intake of each polyamine from all foods and food.

A comparison among three studies which involves total daily polyamine values resulted that the highest contribution to polyamine intake came from putrescine following spermidine and spermine (Table 3). In our study, the contribution to daily intake was from 66.5% putrescine, 23.7% spermidine and 9.8% spermine. When we compare the percentage distribution of polyamines with other studies, putrescine was the highest and spermine was the lowest contributor of daily polyamine intake in the Turkish population.

**Table 3:** Total daily intake and percentage distribution of polyamines.

Total Daily Intake of Polyamines (nmol/person/day)				Distribution of Polyamines %			Reference
Putrescine	Spermidine	Spermine	Total Polyamine	Putrescine	Spermidine	Spermine	
211,910	86,959	54,704	353,573	59.9	24.6	15.5	[35]
159,133	54,697	35,698	249,528	63.8	21.9	14.3	[36]
93,057	33,122	13,685	135,899	66.5	23.7	9.8	Present study

## 4. Discussion

In recent years, there has been considerable interest in the influence of ingested polyamines due to their possible functions on cell growth, maintenance and function. Up to date, several research papers were published on the effect of dietary polyamines in health and diseases and on daily consumption of those active bioamines. Multiple abnormalities in the control of polyamine metabolism were shown to be implicated in several pathological processes [37]. In a review by Novotorski *et al.* it was underlined that the polyamine pathway is a rational target for chemoprevention and chemotherapeutics [38]. Although dietary polyamines have many functional and physiological impacts, their contents in foods are variable. Cooking [21], storage conditions and time [39,40,41], seasonal variations [42], household recipes [43] and agricultural conditions [44] have to be taken into consideration when polyamine concentrations in foods were analyzed. Moreover, tremendous variation in polyamine values in fermented products such as sausages and cheeses occur due to differences in the fermentation process [17,45]. Analytical methods including ion exchange, thin-layer and high performance liquid chromatography also showed some variation even in the same type of food [13,46]. Seasonal variation may affect the polyamine concentrations in foods. In our study, we made the food consumption questionnaire in between July and October. Therefore, total polyamine intake rose from summertime vegetables and fruits such as tomato, cucumber, green pepper, eggplant, melon, watermelon, apple, peach rather than winter vegetables and fruits such as cauliflower, broccoli, celery, and citrus fruits. Indeed, most of the studies do not mention the season when the samples were collected for polyamine analyses.

The analyses of polyamine concentration in foods and the total amount of polyamine intake for USA [36,47], UK [1,16], Norway [17], Germany [21], Japan [9,13,14], Sweden [34], Czech Republic [20,18], Spain [26,32], France [15], Netherlands [35] have been reported in several papers. In the present study, we reached the polyamine concentrations of 255 food items from a literature survey, and calculated the mean value for the same foods, decreasing the number to 161. Since we selected the most frequently consumed foods, only 81 food items were included to calculate daily polyamine intake. In previous studies, Zoumass [36] included approximately 117 of the 370 foods from the literature that produced polyamine content data. Atiya Ali [34] developed a database using 241 food items. Their polyamine values were obtained from literature to measure food intake derived from dietary surveys. Nishibori [14] dealt with 102 food items among 1000 foods in the study of the polyamine intakes in foods in Japan.

Country-specific food preferences and preferences for their main dietary sources might result in the differences in daily polyamine intake. Daily intake of mostly consumed foods were depicted by Nishibori [14] as beverages (491 mL/day), cereals (480 g/day), vegetables (257 g/day); by Atiya Ali [34] as lentil soup (250 g/portion), grapefruit juice (200 mL/portion), orange juice (200 mL/portion), cooked soybean and red beans (190 g/portion). Zoumas [36] used Fred Hutchinson Cancer Center FFQ programming to calculate the mean nmol/day of polyamine from the samples which was corn (max. putrescine, 902,880 nmol/serving size; max. spermidine, 221,111 nmol/serving size), grapefruit juice, oranges, orange juice, grits, crab, grapefruit, green pea soup (max. spermidine, 36,988 nmol/serving size), pear, peas, lentil soup and chicken breast in serving size. We defined the most frequently consumed three foods in Turkey were black tea (322.2 mL/day), white bread (201.4 g/day) and yoghurt (92.9 g/day). Our results were consistent with a cohort study in Turkey, indicating that tea (92.9%), white bread (88.9%) and yoghurt (55.1%) were among the most frequently consumed foods [12].

Calculation of daily polyamine intake in frequently consumed foods in the Turkish population showed that the top three sources for each polyamine came from ripened cheese, black tea and green pepper for putrescine; from black tea, white bread and melon for spermidine; from white bread, lentil soup and rice for spermine. Our findings were consistent with the previous studies that state a high putrescine level as the main source of polyamine intake. In the present study, the highest contribution to putrescine came from dairy products, to spermidine from wheat products, to spermine from meat and wheat products. However, in Japan and Britain, vegetables were the main source of spermidine; meat and meat products the main source of spermine [14,24]. This could possibly be due to regional variation of food intake and dietary habitats.

The average estimated polyamine intakes for adults in countries including the United Kingdom, Italy, Spain, Finland, Sweden, and the Netherlands [35] were 211,910 nmol/day putrescine, 86,959 nmol/day spermidine, and 54,704 nmol/day spermine. A sudy in the USA by Zoumass [36] reported that the average daily polyamine intake values were 159,133 nmol/day putrescine, 54,697 nmol/day spermidine, and 35,698 nmol/day spermine. Our estimation for daily intake was 93,057 nmol/day putrescine, 33,122 nmol/day spermidine and 13,685 nmol/day spermine. Although the values differ from each other, the percentage distribution of daily polyamine intake resulted in the level of each polyamine having a similar pattern in different populations; from the highest to the lowest, contribution rose from putrescine, spermidine and spermine.

There are several limitations to the present study. It is mainly limited by the scarcity of published polyamine concentration in foods. Second, we had limited information about polyamine content of local foods in Turkey. Therefore, our study is an approximation of the real intake of the Turkish population. Third, the origin of foods and methodology to analyze polyamines vary among studies. Finally, daily intake of foods is substantially associated with seasonal changes.

## 5. Conclusions

This study gives, for the first time, a complete description of the total polyamine intake and the main food contributors of dietary polyamines in Turkish population. The most frequently consumed three foods were black tea (322.2 mL/day), white bread (201.4 g/day) and yoghurt (92.9 g/day), accounting for approximately 50% of total food intake. The main dietary sources for the total polyamine were dairy products (47.32%), vegetables and grains (21.09%) and wheat products (12.75%). The estimation for daily intake were 93,057 nmol/day putrescine, 33,122 nmol/day spermidine and 13,685 nmol/day spermine.

## References

[1] Bardocz S., Grant G., Brown D.S., Ralph A., Pusztai A. (1993). Polyamines in food—Implications for growth and health. J. Nutr. Biochem..

[2] Seiler N., Atanassov C.L., Raul F. (1998). Polyamine metabolism as target for cancer chemoprevention. Int. J. Oncol..

[3] Gerner E.W., Meyskens F.L. (2004). Polyamines and Cancer: Old molecules, new understanding. Nat. Rev. Cancer.

[4] Russell D.H., Levy C.C. (1971). Polyamine accumulation and biosynthesis in amouse L1210 leukemia. Cancer Res..

[5] Soda K. (2011). The mechanisms by which polyamines accelerate tumor spread. J. Exp. Clin. Cancer Res..

[6] Sarhan S., Knodgen B., Seiler N. (1989). The gastrointestinal tract as polyamine source for tumour growth. Anticancer Res..

[7] Deloyer P., Peulen O., Dandrifosse G. (2001). Dietary polyamines and non-neoplastic growth and disease. Eur. J. Gastroenterol. Hepatol..

[8] Das R., Kanungo M.S. (1982). Activity and modulation of ornithine decarboxylase and concentrations of polyamines in various tissues of rats as a function of age. Exp. Gerontol..

[9] Nishimura K., Shiina R., Kashiwagi K., Igarashi K. (2006). Decrease in polyamines with aging and their ingestion from food and drink. J. Biochem..

[10] Binh P.N.T., Soda K., Maruyama C., Kawakami M. (2010). Relationship between food polyamines and gross domestic product in association with longevity in Asian countries. Health.

[11] Silla-Santos M.H. (1996). Biogenic amines: Their importance in foods. Int. J. Food Microbiol..

[12] Türkiye Beslenme ve Sağlık Araştırması 2010 (in Turkish). A Report of 2010 National Nutrition Health and Food Consumption Survey. http://www.sagem.gov.tr/TBSA_Beslenme_Yayini.pdf.

[13] Okamoto A., Sugi E., Koizumi Y., Yanagida F., Udaka S. (1997). Polyamine content of ordinary food stuffs and various fermented foods. Biosci. Biotechnol. Biochem..

[14] Nishibori N., Fujihara S., Akatuki T. (2006). Amounts of polyamines in foods in Japan and intake by Japanese. Food Chem..

[15] Lavizzari T., Teresa Veciana-Nogues M., Bover-Cid S., Marine-Font A., Carmen Vidal-Carou M. (2006). Improved method for the determination of biogenic amines and polyamines in vegetable products by ion-pair high-performance liquid chromatography. J. Chromatogr. A.

[16] Bardocz S., Duguid T.J., Brown D.S., Grant G., Pusztai A., White A., Ralph A. (1995). The importance of dietary polyamines in cell regeneration and growth. Br. J. Nutr..

[17] Eliassen K.A., Reistad R., Risoen U., Ronning H.F. (2002). Dietary polyamines. Food Chem..

[18] Kalac P., Krizek M., Pelikanova T., Langova M., Veskrna O. (2005). Contents of polyamines in selected foods. Food Chem..

[19] Moret S., Smela D., Populin T., Conte L. (2005). A survey on free biogenic amine content of fresh and preserved vegetables. Food Chem..

[20] Kalac P., Svecova S., Pelikanova T. (2002). Levels of biogenic amines in typical vegetable products. Food Chem..

[21] Ziegler W., Hahn M., Wallnofer P.R. (1994). Changes in biogenic amine contents during processing of several plant foods. Deutsche Lebensmittel-Rundschau.

[22] Valsamaki K., Michaelidou A., Polychroniadou A. (2000). Biogenic amine production in Feta cheese. Food Chem..

[23] Farriol M., Venereo Y., Orta X., Company C., Gomez P., Delgado G., Rodríguez R. (2004). Ingestion of antioxidants and polyamines in patients with severe burns. Nutr. Hosp..

[24] Bardocz S. (1995). Polyamines in food and their consequences for food quality and human health. Trends Food Sci. Technol..

[25] Silva C.M.G., Gloria M.B.A. (2002). Bioactive amines in chicken breast and thigh after slaughter and during storage at 4 ± 1 °C and in chicken-based meat products. Food Chem..

[26] Hernandez-Jover T., Izquierdo-Pulido M., Veciana-Nogues M.T., Marine-Font A., Vidal-Carou M.C. (1997). Effect of starter cultures on biogenic amine formation during fermented sausage production. J. Food Prot..

[27] Saaid M., Saad B., Hashim N.H., Ali A.S.M., Saleh M.I. (2009). Determination of biogenic amines in selected Malaysian food. Food Chem..

[28] Durlu-Ozkaya F., Alichanidis E., Litopoulou-Tzanetaki E., Tunail N. (1999). Determination of biogenic amine content of Beyaz cheese and biogenic amine production ability of some lactic acid bacteria. Milchwissenschaft.

[29] Novella-Rodriguez S., Veciana-Nogues M.T., Vidal-Carou M.C. (2000). Biogenic amines and polyamines in milks and cheeses by ionpair high performance liquid chromatography. J. Agric. Food Chem..

[30] Novella-Rodriguez S., Veciana-Nogues M.T., Izquierdo-Pulido M., Vidal-Carou M.C. (2003). Distribution of biogenic amines and polyamines in cheese. J. Food Sci..

[31] Fernandez M., Linares D.M., del Rio B., Ladero V., Álvarez M.A. (2007). HPLC quantification of biogenic amines in cheeses: Correlation with PCR-detection of tyramine-producing microorganism. J. Dairy Res..

[32] Novella-Rodriguez S.N., Veciana-Nogues M.T., Roig-Sagues A.X., Trujillo-Mesa A.J., Vidal- Carou M.C. (2004). Evaluation of biogenic amines and microbial counts throughout the ripening of goat cheeses from pasteurized and raw milk. J. Dairy Res..

[33] Stratton J.E., Hutkins R.W., Taylor S.L. (1991). Biogenic amines in cheese and other fermented food: A review. J. Food Prot..

[34] Ali M.A., Poortvliet E., Stromberg R., Yngve A. (2011). Polyamines in foods: Development of a food database. Food Nutr. Res..

[35] Ralph A., Englyst K., Bardocz S., Bardócz S., White A. (1999). Polyamine content of the human diet. Polyamines in Health and Nutrition.

[36] Zoumas-Morse C., Rock C.L., Quintana E.L., Neuhouser M.L., Gerner E.W., Meyskens F.L. (2007). Development of a polyamine database for assessing dietary intake. J. Am. Diet. Assoc..

[37] Moinard C., Cynober L., de Bandt J.L. (2005). Polyamines: Metabolism and implications in human diseases. Clin. Nutr..

[38] Nowotarski S.L., Woster P.M., Casero R.A. (2013). Polyamines and cancer: Implications for chemotherapy and chemoprevention. Expert Rev. Mol. Med..

[39] Valero D., Martinez-Romero D., Serrano M. (2002). The role of polyamines in the improvement of the shelf life of fruit. Trends Food Sci. Technol..

[40] Kozova M., Kalac P., Pelikanova T. (2009). Contents of biologically active polyamines in chicken meat, liver, heart and skin after slaughter and their changes during meat storage and cooking. Food Chem..

[41] Veciana-Nogues M.T., Mariné-Font A., Vidal-Carou M.C. (1997). Biogenic amines in fresh and canned tuna. Effects of canning on biogenic amine contents. J. Agric. Food Chem..

[42] b>Wang S.Y., Faust M. (1993). Comparison of seasonal growth and polyamine content in shoots of orchard-grown standard and genetic dwarf apple trees. Physiol. Plant..

[43] Working Group on Monitoring Scottish Dietary Targets Workshop A Short Review of Dietary Assessment Methods Used in National and Scottish Research Studies Briefing Paper Prepared for: September 2003. http://multimedia.food.gov.uk/multimedia/pdfs/scotdietassessmethods.pdf.

[44] Motyl T., Ploszaj T., Wojtasik A., Kukulska W., Podgurniak M. (1995). Polyamines in cow’s and sow’s milk. Comp. Biochem. Physiol. B Biochem. Mol. Biol..

[45] Kalac P., Spicka J., Krizek M., Steidlova S., Pelikanova T. (1999). Concentrations of seven biogenic amines in sauerkraut. Food Chem..

[46] Slocum R.D., Flores H.E., Galston A.W., Weinstein L.H. (1989). Improved method for HPLC analysis of polyamines, agmatine and aromatic monuamines in plant tissue. Plant Physiol..

[47] Weiger T.M., Aichberger S., Wallace H.M. A comparison of dietary polyamine uptake by humans in Europe, Asia and the USA. Proceedings of the COST 922 Workshop Health Implications of Dietary Amines.

